# Transplantation of Induced Pluripotent Stem Cells Alleviates Cerebral Inflammation and Neural Damage in Hemorrhagic Stroke

**DOI:** 10.1371/journal.pone.0129881

**Published:** 2015-06-18

**Authors:** Jie Qin, Xun Ma, Haiyun Qi, Bo Song, Yanlin Wang, Xuejun Wen, Qing Mei Wang, Shilei Sun, Yusheng Li, Rui Zhang, Xinjing Liu, Haiman Hou, Guangming Gong, Yuming Xu

**Affiliations:** 1 Third Department of Neurology and Key Disciplines Laboratory of Clinical Medicine of Henan Province, The First Affiliated Hospital of Zhengzhou University, Zhengzhou, Henan 450052, P. R. China; 2 Department of Chemical and Life Science Engineering, Institute for Engineering and Medicine, Virginia Commonwealth University, Richmond, VA 23284, United States of America; 3 Department of Physical Medicine and Rehabilitation, Spaulding Rehabilitation Hospital, Harvard Medical School, Boston, MA 02114, United States of America; 4 Department of Microbiology and Immunology, College of Basic Medical Sciences, Zhengzhou University, Zhengzhou, Henan 450001, P. R. China; National University of Singapore, SINGAPORE

## Abstract

**Background:**

Little is known about the effects of induced pluripotent stem cell (iPSC) treatment on acute cerebral inflammation and injuries after intracerebral hemorrhage (ICH), though they have shown promising therapeutic potentials in ischemic stoke.

**Methods:**

An ICH model was established by stereotactic injection of collagenase VII into the left striatum of male Sprague-Dawley (SD) rats. Six hours later, ICH rats were randomly divided into two groups and received intracerebrally 10 μl of PBS with or without 1×10^6^ of iPSCs. Subsequently, neural function of all ICH rats was assessed at days 1, 3, 7, 14, 28 and 42 after ICH. Inflammatory cells, cytokines and neural apoptosis in the rats’ perihematomal regions, and brain water content were determined on day 2 or 3 post ICH. iPSC differentiation was determined on day 28 post ICH. Nissl^+^ cells and glial fibrillary acidic protein (GFAP)^+^ cells in the perihematoma and the survival rates of rats in two groups were determined on post-ICH day 42.

**Results:**

Compared with control animals, iPSCs treatment not only improved neurological function and survival rate, but also resulted in fewer intracephalic infiltrations of neutrophils and microglia, along with decreased interleukin (IL)-1β, IL-6 and tumour necrosis factor-alpha (TNF-α), and increased IL-10 in the perihematomal tissues of ICH rats. Furthermore, brain oedema formation, apoptosis, injured neurons and glial scar formation were decreased in iPSCs-transplanted rats.

**Conclusions:**

Our findings indicate that iPSCs transplantation attenuate cerebral inflammatory reactions and neural injuries after ICH, and suggests that multiple mechanisms including inflammation modulation, neuroprotection and functional recovery might be involved simultaneously in the therapeutic benefit of iPSC treatment against hemorrhagic stroke.

## Introduction

Intracerebral haemorrhage (ICH) is a serious stroke subtype associated with high morbidity, mortality, disability and recurrence rate [1–3]. ICH-induced primary injury is mainly caused by the hematoma formation and its expansion within brain parenchyma that resulting in the damage of brain cell structure and the increase of intracranial pressure. ICH-induced secondary injury is caused by three intertwined degenerative cascades including inflammation, red cell lyses and thrombin production, which induce severe neural dysfunctions by the formation of cerebral edema, neuronal apoptosis and degeneration[1, 3, 4]. Accumulating evidence has shown that cerebral inflammatory responses, including the infiltration of inflammatory cells such as neutrophils and mononuclear-macrophages, microglial activation and the secretion of inflammatory-related cytokines, play a crucial role in all phases of the ICH-induced secondary brain damages. This data suggests that various inflammatory pathways involving specific mediators and inflammatory cells may be as promising therapeutic targets for treating ICH [1, 3–8]. Currently there is limited available effective treatment against inflammatory reaction, which may protect neural cells from ICH-induced injuries [1–4, 9].

Stem cell-based approaches have already developed into effective therapies to restore function in experimental ICH [1, 5, 10–15]. Induced pluripotent stem cells (iPSCs) are stem cell populations generated from adult somatic cells through reprogramming by transcription factors. These cells are regarded as promising candidates for clinical cell therapies without the ethical, immunogenetic and low quantity problems [13, 16]. Thus, reprogramming of human various somatic cells into iPSCs is one of the most perspective approaches to produce sufficient patient-specific cells for autologous transplantation[16, 17]. Advances in iPSCs technologies, including improvements in the ease and efficiency of generating iPSCs and iPSCs-derived neural cells, have resulted in increased adoption of these cells in treating stroke[18, 19]. Recent studies have indicated that transplantation of iPSCs and iPSCs-derived cells holds tremendous potential for the treatments of ischemic stroke[15, 17, 20], and suggested that it could reduce the inflammatory damages in this [15] and other animal models of human diseases[21, 22]. Furthermore, a recent study suggests that iPSCs have more potent immunomodulatory properties compared with bone marrow-derived mesenchymal stromal cells (MSCs) [23]. We have also shown that iPSCs treatment can promote improved recovery of persistent neurological dysfunctions in an experimental ICH model [24]. However, it remains unclear whether iPSC treatment has possible anti-inflammation potential on the innate inflammatory response and inflammatory-induced neural injuries after acute hemorrhagic stroke[7]. In this study, we therefore aim to explore the therapeutic effects of iPSC transplantation on acute cerebral inflammation, neural damage and recovery in a rat collagenase-induced ICH model. These findings may improve the limited treatment strategies currently available for hemorrhagic stroke.

## Materials and Methods

### The cultivation of rat iPSCs

The rat iPSCs line used in this study was provided by Dr. Lei Xiao[25]. T25 Corning flasks were incubated with 0.1% gelatin for 30 min. at 37°C, then murine embryonic fibroblasts (MEFs, 5×10^4^ cells/cm^2^) were cultured in fibroblasts medium (Dulbecco's modified Eagle's medium, DMEM) containing 10% fetal bovine serum (FBS), and 100 IU/ml penicillin/streptomycin (all from Invitrogen except FBS from Hyclone) two days before plating rat iPSCs. iPSCs were washed and centrifuged (1000 rpm×5min.) in a 15 ml centrifuge tube containing 5 ml iPSCs culture medium twice and then plated on confluent MEF feeder in iPSCs culture medium containing Knockout DMEM supplemented with 10% Knockout Serum Replacement (KSR), 1 mM glutamine, 1% NEAAs, and 0.1 mM β-mercaptoethanol (all from Invitrogen). After two days of culture, the iPSCs formed clones on MEFs. Then the iPSCs were passaged in the medium every 3 to 5 days. Medium was changed every other day. Alkaline phosphatase (AP) staining was conducted to identify these iPSCs clones as previously described [13].

### Induction of rat ICH model

This study was approved by the Ethics Committee of Zhengzhou University, Zhengzhou, China (ECZUC). All animal experimental procedures in this study were complied with the institutional guide for the care and use of laboratory animals and carried out under the approval of ECZUC. A total of 152 adult male Sprague-Dawley (SD) rats (250–280 g) were used in our experiments. All rats were 7–8 weeks old and purchased from the Animal Center of Henan province, China. These rats were raised in isolated cages with independent ventilation, living in a 12-hour light-dark cycle and with free access to food and water in a constant temperature (22 ± 2°C). To generate experimental ICH, the rats were stabilized in the stereotactic frame (Narishige SN-3, Tokyo, Japan) in a prone position after being anesthetized with 1% ketamine (30 mg/kg, I.P., Sigma–Aldrich). A midline incision was made through the scalp to expose the skull and then a 1 mm × 1 mm craniectomy was performed. Subsequently, a stereotactically guided needle was placed into the left striatum (0.2 mm posterior and 3.0 mm lateral to the bregma, 6.0 mm depth below the surface of the skull). Ten μL bacterial collagenase VII (Sigma–Aldrich, 0.25 U/μl) was injected using a 10 μl microsyringe at a steady infusion rate of 1 μl/min. Then the wound was cleaned and sutured [10, 13]. The sham rats underwent the same procedure except an equivalent amount of sterile saline was injected instead of collagenase VII (Table 1).

**Table 1:** The characteristics of all rats in each group.

Groups	Sham	PBS	iPSCs
Total	30	63	59
For observing survival rate	18	18	18
Survived rats at day 42 post ICH	17	12	15
Survival rate (= Survived rats/18*%)	94.44	66.67	83.33

### The labeling and implantation of iPSCs

Before the transplantated, iPSCs were labeled with CM-Dil (Invitrogen) following manufacturer’s instructions. Briefly, iPSCs were incubated in culture medium containing CM-Dil (4 ug/ml final concentration) for 30 min. at 37°C. Then the the culture was washed twice with 37°C PBS. Finally, labeled cells were resuspended in PBS at a concentration of 1×10^5^/μl for cell transplantation.

Six hours after inducing ICH, all ICH rats were randomly divided into two groups, the phosphate buffer saline (PBS) control group (PBS only) and the iPSC treatment group (total 1×10^6^ cells in the same amount of PBS). Ten μL PBS with or without CM-Dil-labeled iPSCs was injected into the brain via the original needle tract at the depth of 3.5 mm relative to the bregma, 2.5 mm upon the hemorrhage lesion according to a previous report [26]. After infusion of iPSCs or PBS, the needle was retained for 10 min. and then removed slowly to prevent backflow. Then the wound was sutured after cleaning.

### Behavioral test

Modified limb placing test (MLPT) was performed to evaluate the neurological functions of all ICH rats (n = 8/group) according to previous reports by two blinded investigators independently at days 0, 1, 3, 7, 14, 28 and 42 post ICH[5, 10].

### Measurement of brain water content

Brain water content was measured at day 3 after ICH by a blinded investigator using standard wet–dry method[5, 27]. Twelve rats (n = 6/group) were anesthetized and sacrificed via decapitation. The rat brain was removed immediately and divided along the midline into haemorrhagic hemisphere and contralateral (non-hemorrhagic) hemisphere. Then a single 4-mm section was cut centered around the hematoma. The brain sections were immediately weighed on an electronic analytical balance (Mettler Toledo AL104) to obtain wet weights, and then dried in an oven (CIMO DHG-914385-Ⅲ) at 100°C for 24 hours. to obtain dry weights. Water content was calculated as [(wet weight-dry weight)/wet weight] × 100%.

### Hematein-Eosin (HE) staining and Nissl staining

At day 3 or day 42 after ICH, rats (n = 6/time point/group) were reanesthetized and perfused through the heart with 150 ml cold saline and 150 ml 4% paraformaldehyde in 0.1 M PBS. After 4 hours of fixation in 4% paraformaldehyde, the brains were dehydrated with 30% sucrose for 24 hours at 4°C. Then the tissues surrounding the hematoma were cut into consecutive coronal sections with the thickness of 10 μm on a cryostat (Leica) after being embedded in optimum cutting temperature compound (OCT).

Paraffin-embedded brains were cut into 3 μm coronal sections from the core of hematoma after being fixed with 4% paraformaldehyde for 24 hours. For HE coloration at day 3 post ICH, nuclei were stained with hematoxylin for 3 min. after dehydration in gradient ethanol solution, and differentiated with 0.3% acid alcohol, then stained with eosin solution for 3–5 min. For Nissl staining, sufficiently deparaffinized sections taken at day 42 post ICH were hydrated and stained in warmed 1% toluidine blue solution for 20 min. After rinsing with ultrapure water, they were dehydrated and mounted with permanent mounting medium. Both HE and Nissl staining sections were observed using a light microscope (Leica). For Nissl^+^ cell counting, five non-overlapping 400 × images were obtained randomly from each slice. The percentages of Nissl^+^ cells without shrunken and physalides in all Nissl^+^ cells were counted per image and their mean were calculated by two individuals blinded to the experimental conditions.

### Immunostaining

OCT-embedded, 10 μm coronal sections of hematoma were immunostained with primary antibodies specific for myeloperoxidase (MPO), CD11b, activated caspase-3, NeuN (all diluted at 1:200 and from Abcam) and GFAP (1:200, Santa Cruz) to identify cerebral neutrophils, microglia, apoptotic cells, neuron and astrocytes around the hematoma, respectively. Sections were incubated with primary antibodies for overnight at 4°C, and secondary antibodies, Dylight488, Alexa Fluor 647, or Cy3-conjugated AffiniPure goat anti-rabbit/mouse IgG (H+L) (1:1000, Jackson ImmunoResearch) for 2 hours at room temperature. 4ˊ, 6-diamidino-2-phenylindole (DAPI) was used for nuclear counterstaining. Negative control sections were stained in an identical manner except that the primary antibodies were omitted. For cell counting, five non-overlapping 400 × images (310μm × 310μm) were obtained randomly from each tissue slice cutting from the perihematoma areas of per rat (total 5 sections from each rat were used for primary antibody staining) using a confocal laser scanning microscope (ZEISS). Four individuals blinded to the experimental conditions counted the number of positive cells per image. The thickness of glial scar was investigated by measuring the thickness of GFAP-positive cell staining areas.

### Real-time PCR

At day two post-ICH, after being anesthetized, rats were sacrificed (n = 6/group). Then the tissues around the hematoma were removed and cryopreserved in liquid nitrogen immediately. The total RNA of perihematoma tissues was extracted using the SV total RNA Isolation System and the first-strand cDNA was synthesized by Reverse Transcription System (all from Promega) according to the manufacturer’s protocols. Real-time quantitative PCR was performed using GoTaq qPCR Master Mix on ABI PrismTM 7500 Fast System (Applied Biosystems) according to the manufacturer’s protocol. Amplification of glyceraldehyde phosphate dehydrogenase (GAPDH) served as internal-control for sample loading. The sequences of all primers for real-time PCR (Sangon, China) are: GAPDH: 5’-TGC ACC ACC ACC TGC TTA GC-3’(sense), 5’-GGC ATG GAC TGT GGT CAT GAG-3’(antisense); TNF-α: 5’-CAT GGA TCT CAA AGA CAA CCAA-3’(sense), 5’-CTC CTG GTA TGA AAT GGC AAAT-3’(antisense); IL-6: 5’-GAC AGC CAC TCA CCT CTT CAG-3’(sense), 5’-CAT CCA TCT TTT TCA GCC ATC-3’(antisense); IL-1β: 5’-CTT CAA ATC TCA CAG CAG CATC-3’(sense), 5’-GCT GTC TAA TGG GAA CAT CACA-3’(antisense) and IL-10: 5’-CAG TCA GCC AGA CCC ACA T-3’(sense), 5’-GGC AAC CCA AGT AAC CCT-3’(antisense). The results of real-time PCR were analyses using 2-ΔΔCt method as previous report[28]. 2-ΔΔCT = [(CT gene of interest-CT internal control) sample A—(C_T_ gene of interest—C_T_ internal control) sample B)].

### ELISA

At day two post ICH, rats were sacrificed after being anesthetized (n = 6/group). Brain tissue was rinsed with cold PBS (0.01M, PH = 4) to remove blood. Tissue was then minced into small pieces and homogenized in PBS on ice. Brain homogenates were ollected and centrifugated for 5 min. at 5000 g. The supernate was tested for rat IL-1β, IL-6, TNF-α and IL-10 using ELISA Kits (Elabscience) according to the manufacturer’s protocol.

### Statistical Analysis

All data are presented as Means ± SEM. For comparisons between two groups, data were analyzed by two-tailed Student’s t-test with normality (Kolmogorov-Smirnov test, *P*>0.1) or Mann-whitney U test. For comparisons among three groups, data were analyzed by Bonferroni analysis of variance (ANOVA). The MLPT scores were analyzed by repeated measure analysis. A two-tailed *P*-value of < 0.05 was considered statistically significant.

## Results

### Transplanted iPSCs differentiate into neural cells in the ICH-injured brain

We previously reported that the transplantation of human iPSCs at 24 hours. after ICH can give rise to better functional recovery in rats with experimental ICH, and suggested that cell replacement and/or neurotrophic action may be involved in the therapeutic effects of iPSCs treatment for ICH[24]. In this study, we tested the hypothesis that anti-inflammation and neuroprotective effects may play a role in the improved functional recovery in iPSCs-grafted animals with experimental ICH. For this reason we used a rat iPSCs line, instead of more commonly used human or mouse iPSCs, to treat rat experimental ICH models with no need for immunosuppression and no more than minimal immune interferences.

**Fig 1:**
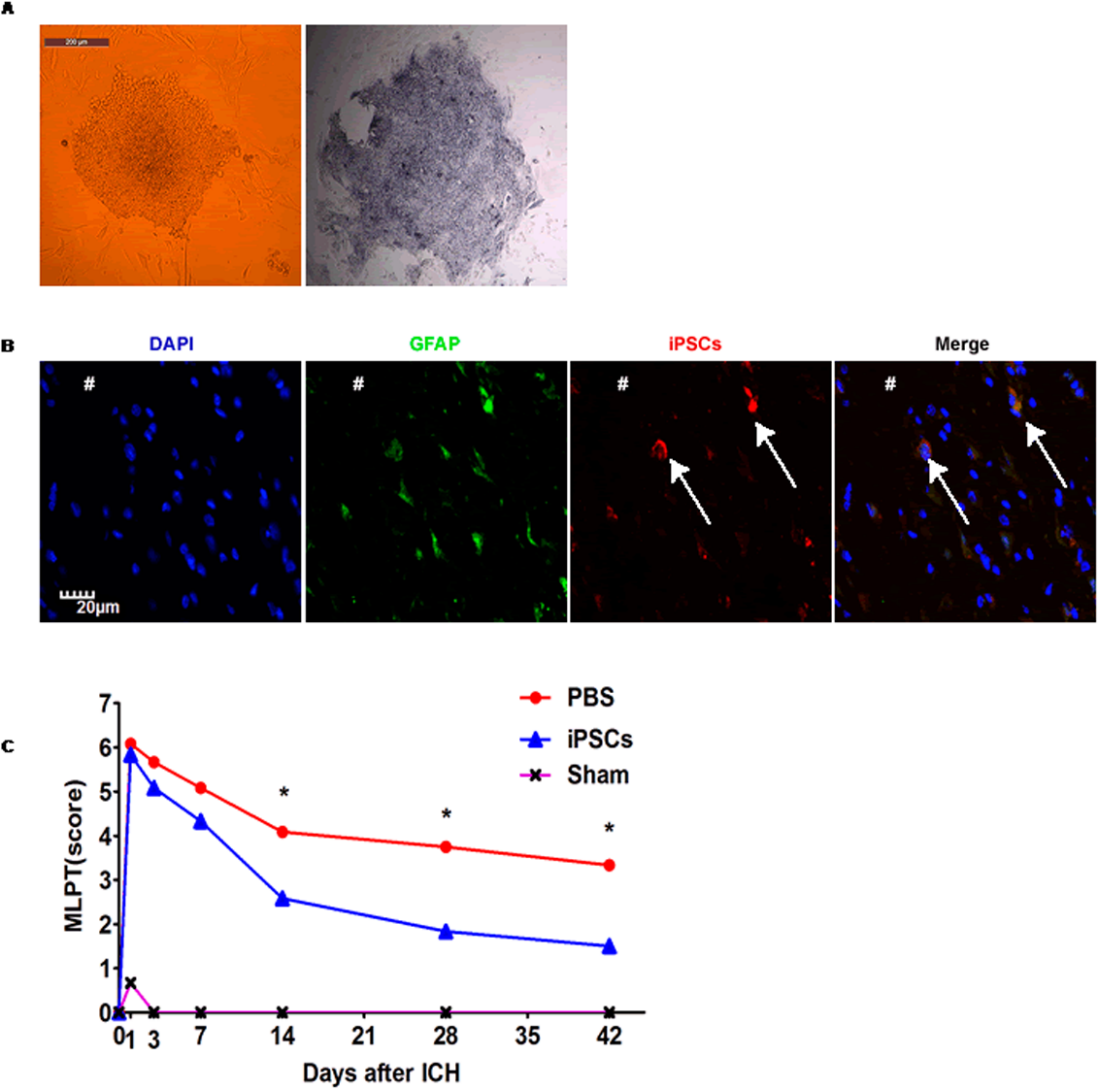
Transplanted iPSCs differentiate into GFAP^+^ neural cells in the perihematomal regions and enhance the functional recovery of ICH rats. (A) Passaged iPSCs displayed typical colonies (left) and positive AP (right; bar=200 μm). (B) Immunofluorescence for GFAP showed the CM-Dil labeled iPSCs within the perihematomal regions differentiated into neural cells at day 28 post ICH. The # sign indicates hematoma areas. Bar=20 μm. (C) Changes in scores of MLPT in rats of 3 groups (Sham; PBS; PBS+iPSCs) over time after treatment. Shown are the mean values with SEM from 8 rats for each group. *P<0.05 for differences between PBS and PBS+iPSCs groups at days 14 to 42 post ICH.

Rat iPSCs formed typical clones (Fig 1A left) and expressed AP (Fig 1A right). In order to determine the neural differentiation of transplanted iPSCs in the ICH brain, the CM-Dil^+^GFAP^+^ cells were detected to identify neural cells originating from the graft. With a multicolor fluorescence assay we found that iPSCs differentiated into GFAP^+^ neural cells in rat brain at day 28 post ICH (Fig 1B).

### iPSC treatment improves neural functions and survival rate of ICH rats

The neural functions and survival rate of all rats were observed to determine the protective effects of iPSCs transplantation on acute ICH-induced injuries. The rats in the iPSC-transplanted group showed continuous improvement of motor function indexed by MLPT scores as compared to vehicle-injected animals from day 14 to day 42 post ICH induction (*P*<0.05, respectively), while sham ICH rats did not show any neurological deficits (Fig 1C). Furthermore, the survival rate of rats in iPSC group (83.33%) was improved compared with that of rats in PBS group (66.67%) at day 42 post ICH (Table 1).

### iPSC treatment significantly reduces the number of inflammatory cells and the expression of IL-1β, IL-6 and TNF-α but not IL-10 in the perihematomal areas

To explore the anti-inflammatory effects of iPSC transplantation for hemorrhagic stroke, we investigated the counts of acute inflammatory cells including neutrophils and microglia. We also measured expression levels the expressing levels of inflammatory cytokines including TNF-α, IL-1β, and IL-6 and anti-inflammatory cytokine IL-10 in the perihematomal areas of iPSC-transplanted ICH rats.

**Fig 2:**
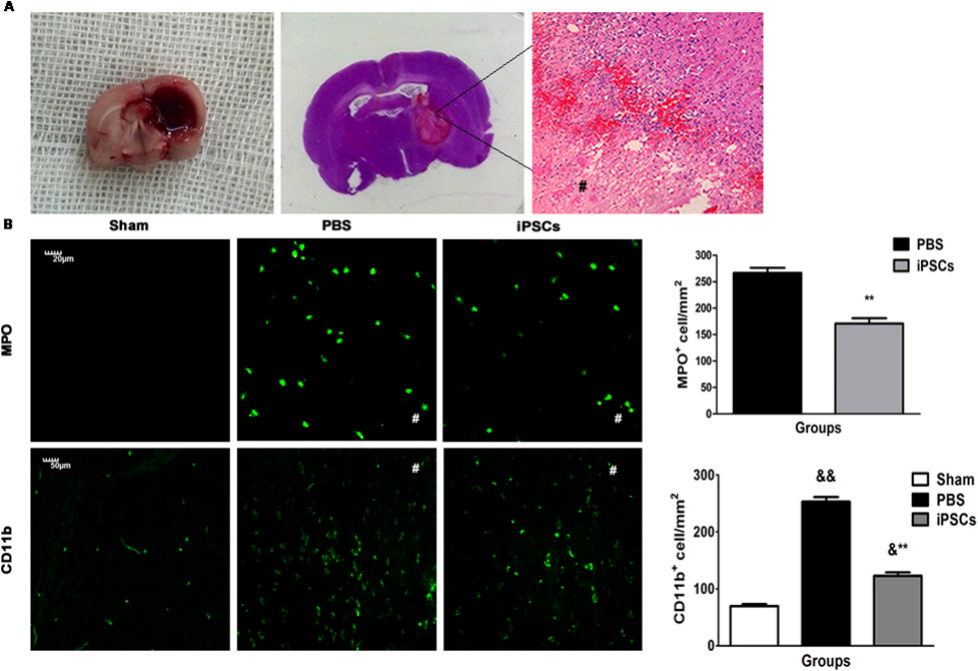
Reduction of cerebral inflammatory cells in iPSC-grafted rats. (A) Representative image of the collagenase-induced hemorrhagic lesion (left). HE staining showed that infiltration of inflammatory cells in the perihematoma areas (middle and right; The # sign indicates hematomal area). (B) The counts of MPO^**+**^ and CD11b^**+**^ inflammatory cells at day 3 post ICH. Immunostaining shows representative images of MPO^**+**^ cells (top panel) and CD11b^**+**^ cells (bottom panel) (Sham; PBS; PBS+iPSCs). Histograms on the right show changes in the counts of MPO^**+**^ and CD11b^**+**^ cells at day 3 post ICH. Counts of MPO^**+**^ and CD11b^**+**^ inflammatory cells in perihematoma areas were significantly decreased in iPSCs group compared to the PBS group, while there were no MPO^**+**^ inflammatory cell and very few CD11b^**+**^ inflammatory cells in Sham group. Data are mean values with SEM. MPO^**+**^ cells, **P<0.01 for differences between PBS and PBS+iPSCs groups at day 3 post ICH. CD11b^**+**^ cells, &&*P*<0.01 or &*P*<0.05 for differences between Sham and PBS groups or PBS+iPSCs groups; **P<0.01 for differences between PBS and PBS+iPSCs groups at day 3 post ICH (n = 6/group; The # signs indicate hematomal areas).

On day 3 post ICH, HE staining of brain tissue sections showed a large amount of inflammatory cells distributing in the areas around hematoma (Fig 2A), though there was no difference in the volume of hematoma in two groups (data not shown). Immunostaining assays for identifying neutrophils and microglia were conducted to accurately analyze the numbers and subtypes of inflammatory cells in the perihematoma. Stereological quantification of the total number of inflammatory cells revealed that iPSC transplantation resulted in a significant decrease in the inflammatory cells accumulation, including MPO^+^ neutrophils and CD11b^+^ microglia in the perihematomal areas, compared with that of rats in the PBS group (MPO^+^ neutrophils: Sham group, 0 cells/mm^2^; PBS group, 266.57±9.82 cells/mm^2^ and iPSCs group, 170±10.17 cells/mm^2^; PBS group *versus* iPSCs group, *P*<0.01; CD11b^+^ microglia: Sham group, 74.72±3.38 cells/mm^2^; PBS group, 256.74±7.58 cells/mm^2^; iPSCs group, 136.93±6.24 cells/mm^2^. Sham group *versus* PBS group, *P*<0.01; Sham group *versus* iPSCs group, *P*<0.05; PBS group *versus* iPSCs group, *P*<0.01) (Fig 2B).

**Fig 3:**
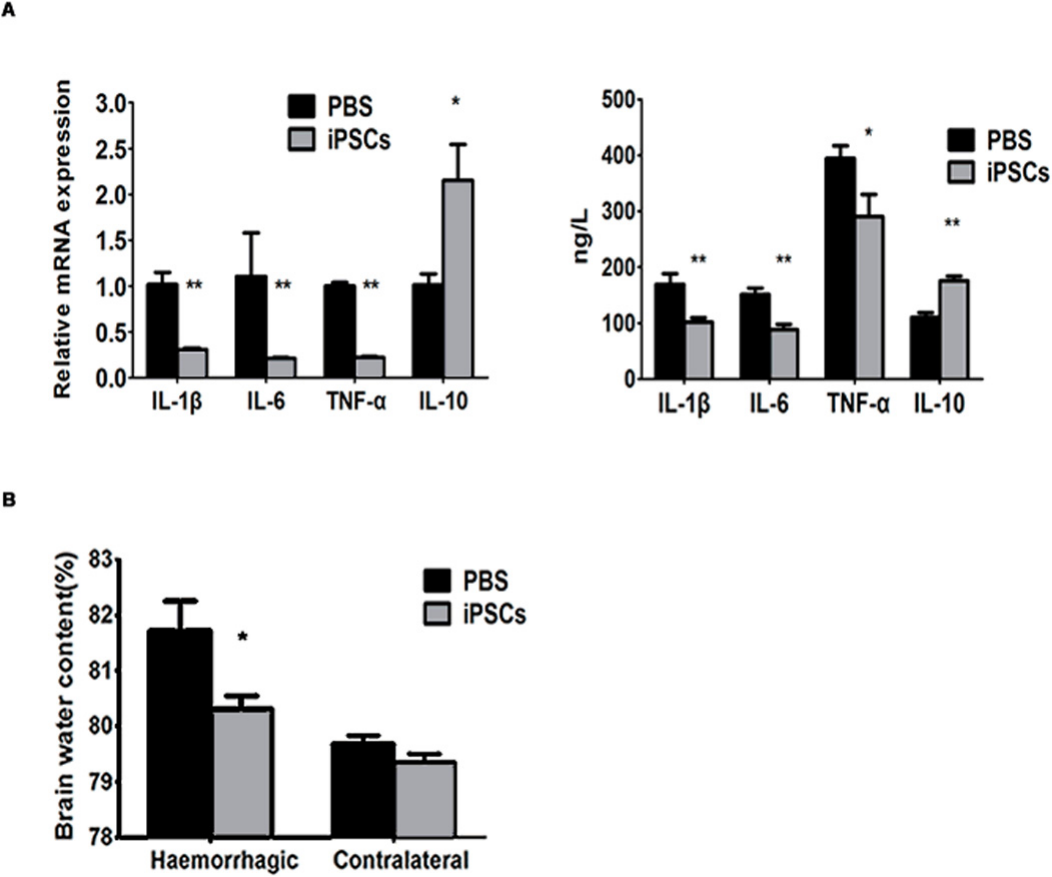
Changes of cerebral cytokines and encephaledema in iPSC-grafted rats. (A left) Relative mRNA levels of IL-1β IL-6 and TNF-α at day 2 post ICH in the perihematoma were decreased in iPSCs group compared with PBS group (n = 6/group; **P<0.01 compared with PBS group), but IL-10 increased (n = 6/group; *P<0.05 compared with PBS group). Data are mean values with SEM. (A right) Protein levels measured by ELISA of IL-1β IL-6 and TNF-α at day 2 post ICH in the perihematoma were also decreased in iPSCs group compared with PBS group (n = 6/group; iPSCs group *versus* PBS group: IL-1β and IL-6, **P<0.01 respectively, TNF-α, *P<0.05), but IL-10 increased (n = 6/group; **P<0.01 compared with PBS group). Data are mean values with SEM. (B) Brain water content in the hemorrhagic hemisphere of rats in iPSCs group was significantly lower than PBS group (n = 6/group; *P<0.05 compared with PBS group), while the non-hemorrhagic hemisphere didn’t show any difference between two groups (*P* = 0.11 compared with PBS group). Data are mean values with SEM.

In addition to inflammatory cells, the inflammatory cytokines including TNF-α, IL-1β, IL-6, and IL-10 were measured in the perihematomal areas at day 2 after ICH by RT-PCR and ELISA test. We found that the RNA and protein levels of IL-1β, IL-6, and TNF-α were reduced significantly in the brains of iPSC-transplanted rats (RNA: IL-1β, IL-6, and TNF-α, *P*<0.01, respectively. Protein: IL-1β and IL-6, *P*<0.01, respectively; TNF-α, *P*<0.05). In contrast, IL-10 expression increased (RNA, *P*<0.05 and protein, *P*<0.01), compared with that of the PBS group (Fig 3A).

### iPSC treatment reduces brain water content

The analysis of brain water content was conducted at day 3 post ICH to identify the effect of iPSC treatment on cerebral edema. The mean of brain water content of haemorrhagic hemispheres was significantly different between the PBS group (81.71±0.54%) and the iPSC group (80.31±0.24%) (*P*<0.05). However, there was no difference in the mean of brain water content of contralateral (non-hemorrhagic) hemispheres between these two groups (PBS group = 79.69±0.14% and iPSC group = 79.35±0.15%, *P* = 0.11) (Fig 3B).

### iPSC treatment reduces apoptotic neural cells in the perihematomal areas

**Fig 4:**
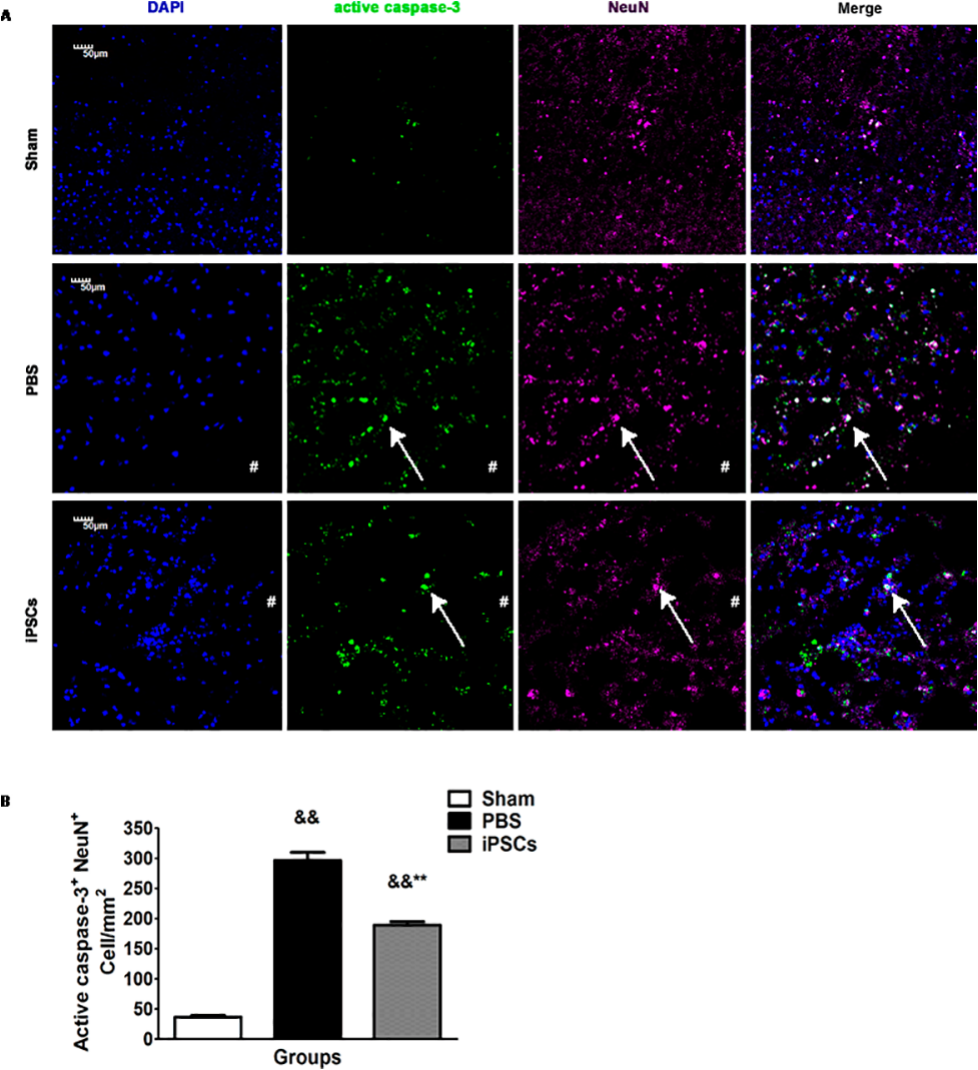
Reduction of cerebral apoptosis of neural cells in iPSC-grafted rats. (A) Representative staining for activated caspase-3^**+**^NeuN^**+**^ cells show changes in apoptosis of neural cells (Sham; PBS; PBS+iPSCs). Bar=50 μm (B) Histograms show changes in the counts of caspase-3^**+**^NeuN^**+**^ cells at day 3 post ICH. Data are mean values with SEM. The counts of the caspase-3^**+**^NeuN^**+**^ cells around the hematoma in iPSCs group were lower than that in PBS group (n = 6/group; &&*P*<0.01 respectively, compared with Sham group; **P<0.01 compared with PBS group).

To determine the neuroprotective effects of iPSC treatment for acute ICH, apoptotic neural cells in the perihematomal areas were investigated on day 3 post ICH by using immunostaining assay for caspase-3 and NeuN double-positive cells. As displayed in Fig 4, the numbers of caspase-3^+^NeuN^+^ cells in the iPSC group (189.15±5.76 cells/mm^2^) were much fewer than that of the PBS group (296.39±13.58 cells/mm^2^, *P*<0.01). (Fig 4).

### iPSC treatment reduces neuronal damage and glial thickness in the perihematomal areas

**Fig 5:**
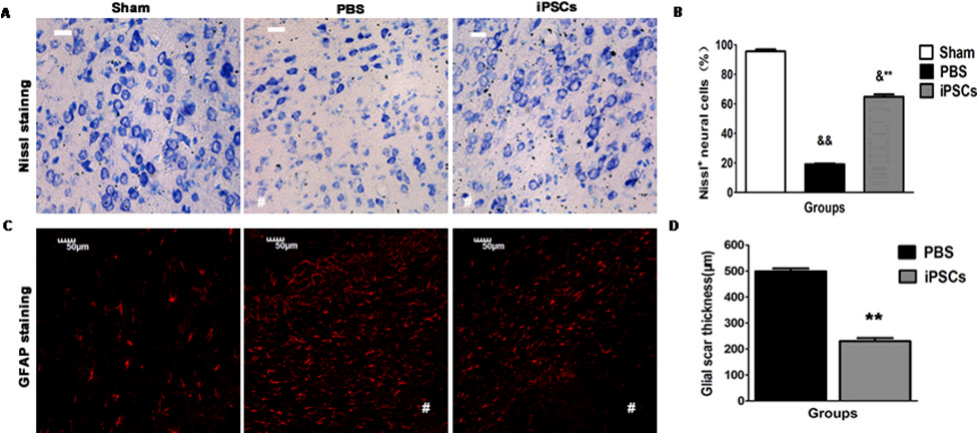
Reduction of damaged neurons and glial scar in the perihematomal areas. (A) Representative Nissl staining for each group (Sham; PBS; PBS+iPSCs) at day 42 post ICH. (B) Histograms show changes in percentages of Nissl^**+**^ cells at day 42 post ICH. Data are mean values with SEM. There was less damage to Nissl bodies and nucleus within neural cells in the iPSC group compared with that in the PBS group, while it has hardly any damage to Nissl bodies and nucleus in Sham group (A and B; n = 6/group; &&*P*<0.01 compared with Sham group; &*P*<0.05 compared with Sham group; **P<0.01 compared with PBS group. The # signs indicate hematomal areas. Bar=20 μm). (C) Representative immunostaining for GFAP^**+**^ cells of one representative rat of 3 groups (Sham; PBS; PBS+iPSCs) at day 42 post ICH. (D) Histograms show changes in the thickness of glial scar through measuring GFAP^**+**^ cells at day 42 post ICH. Shown are the mean values with SEM. The thickness of glial scar in iPSC group is less than that of PBS group (C and D; n = 6/group; **P<0.01 compared with PBS group. The # signs indicate hematomal areas. Bar=50 μm).

ICH leads to neural degeneration via inducing neuron damage and forming glial scars by promoting the glial proliferations in the perihematomal areas. At day 42 post ICH, Nissl staining and measuring the thickness of GFAP^+^ cells were conducted respectively to determine the changes of neural damage and the thickness of glial scars in the perihematomal areas after iPSC transplantation. As shown in Fig 5, the percentage of Nissl^+^ cells without shrunken and physalides in all Nissl^+^ cells in the iPSC group (64.87 ± 1.60%) was much more than that of the PBS group (19.14 ±0.61%), while less than Sham group (95.62±1.34%) (Sham group *versus* PBS group, *P*<0.01; Sham group *versus* iPSCs group, *P*<0.05; PBS group *versus* iPSCs group, *P*<0.01) (Fig 5A and 5B). Furthermore, compared with the PBS group (498±11.58 μm), the glial thickness with GFAP^+^ cells staining areas in the perihematoma was also significantly decreased in the iPSC group (230±11.55 μm) (*P*<0.01) (Fig 5C and 5D).

## Discussion

We previously reported that transplantation of human iPSCs into the stroke-damaged rat striatum can improve the functional recovery in an experimental ICH model and may act through neuronal replacement and boosting neuroprotective factors[24]. However, the *in vivo* mechanism(s) by which grafted iPSCs antagonize ICH–induced injuries is still unclear. To extend our understanding of the treating basis of iPSC transplantation for ICH, we performed the current study and provided further evidences that the anti-inflammatory and neuroprotective effects of iPSCs treatment may play an important role in the functional restoration post acute ICH. Our findings suggest that iPSC-transplanted rats exhibit not only functional improvement and increased survival rate, but also reduced infiltration of inflammatory cells, down-regulated proinflammatory cytokines, and up-regulated anti-inflammatory cytokines, as well as attenuated apoptosis, edema, and glial scar in an ICH-damaged brain.

Stem cell-based therapies have great potential to treat human neurological diseases, especially in intractable stroke and degenerative diseases[12, 14, 29–33]. Multiple types of stem cells such as neural stem cells (NSCs) and MSCs have so far shown potent immunomodulatory and neuroprotective properties in the models of human ischemic and hemorrhagic stroke[14, 29, 30, 34–36]. For example, Lee et al. [5] reported that intravenously injected NSCs have neuroprotective and anti-inflammatory actions in hemorrhagic stroke. Though NSCs and MSCs have such promising potentials in treating stroke, the difficulty in obtaining large numbers of cells and poor proliferating ability limit their application for autologous cell transplantation. Much recent research shows that iPSCs reprogrammed from autologous cells have more therapeutic potentials than adult stem cells in treating ischemic stroke [15, 17–20]. One of these studies reported that subdural transplantation of iPSCs in rats with experimental cerebral ischemic could improve their motor function, attenuate inflammation cytokines, increase anti-inflammatory cytokines, and exert neuroprotective effects [15]. Another research reported that human iPSC-derived neural precursor cells (NPCs) can enhance behavioral recovery, reduce stroke-induced inflammatory response, gliosis and apoptosis in rats with ischemic stroke [37]. More importantly, a recent study found out that iPSCs had similar immunogenic properties but more potent immunomodulatory effects than bone marrow-derived MSCs *in vitro*[23]. It is still not clear whether iPSCs have similar characteristics as adult MSCs and NSCs in treating hemorrhagic stroke [24].

In this study, we present the first experimental evidence that rat skin-derived iPSCs can significantly reduce inflammatory infiltrations of neutrophils and microglia around the intracerebral hematoma. Neuroinflammation after ICH is a double-edged sword. On the one hand, to clear degenerative and necrotic neurons and astrocytes post ICH, it requires the infiltration of specific immunocyte subtypes with phagocytic function into the ICH brain. On the other hand, excessive inflammatory responses lead to detrimental effects contributing to secondary brain injuries post ICH [1, 6]. Among infiltrated immunocytes, neutrophils are the earliest immunocyte subtype to infiltrate into the brain after ICH, appear within 4 to 5 hours and peak at day 3 in animal ICH models[5, 7, 9]. Resident microglia cells in the brain are the first non-neuronal cells to respond to ICH-induced damages. They become activated within 1 hour after ICH and peak at 3–7 days [4–6]. Like neutrophils, microglia cells can clear up the hematoma by engulfing degenerative and necrotic cells[7]. Furthermore, they can secret proinflammatory cytokines such as TNF-α, IL-1β and IL-6 to recruit other immunocytes such as neutrophils and lymphocytes, which further aggravate brain injuries[5, 7]. There have been plenty of evidence suggesting that inhibition of microglia cells could benefit the prognosis of ICH [9]. Our observation of decreased infiltrations of neutrophils and microglia following intrastriatal implantation of the iPSCs suggests their immunomodulatory effects on the acute inflammatory response after ICH.

Inflammatory networks contributing to brain damage after ICH involve not only inflammatory cells, but also inflammatoion-related cytokines [6, 9, 38]. It has been indicated that inflammatory cytokines including TNF-α, IL-1β and IL-6 give rise to edema and brain damage post ICH by recruiting neutrophils and lymphocytes infiltrating into brain to destroy the integrity of blood-brain barrier and other brain tissues; however, anti-inflammatory cytokine IL-10 has beneficial effects against the brain damages post ICH[1, 4–7, 38, 39]. We found that TNF-α, IL-1β and IL-6 decreased, while IL-10 elevated significantly in the perihematomal tissues of iPSC-transplanted rats. These results provided further evidence that iPSC grafts are able to not only suppress intracerebral infiltrations of inflammatory cells, but also effectively regulate the production and the secretion of inflammation-related cytokines in the perihematomal regions of ICH-injured brain.

Besides direct anti-inflammatory effects, we have observed that iPSC treatment could mitigate brain edema formation in ICH rats through indirect immunomodulatory properties. As the common end point of multiple pathogenic pathways, edema, resulting in abnormal water accumulation around the hematoma areas leading to increased intracranial pressure, is a predictor of poor outcome post ICH. Cerebral edema is involved in almost every aspect of primary and secondary damages post ICH. Inflammatory reaction is the main cause of vasogenic edema after ICH [1, 4, 5, 38]. It can be inferred that alleviated edema in iPSC-implanted rats may be due to the reduced inflammatory response compared with that of the control rats.

Loss of neurons in ICH-injured brain is a major cause of various stroke-induced neurological deficits. ICH-injured neurons go through apoptosis or necrosis, which are exacerbated by undue inflammatory responses. Indeed, we have found that apoptotic neural cells, neuronal damage and glial thickness were simultaneously reduced along with a decline of inflammatory reactions in the perihematomal areas of the iPSC-treated rats [5, 38]. These results indicate the feasibility of using iPSCs to reduce cerebral inflammation and secondary brain damage after ICH[6].

We also found that iPSCs have the potential to differentiate into neural cells in ICH brain, suggesting that neural replacement was involving in the improved functional recovery post ICH. In addition to improving neural function, another exciting effect of iPSC intervention on the outcome and prognosis of acute ICH is that enhancing ICH rats’ survival rate. Altogether, these results show that iPSCs have multiple roles in exerting beneficial effects on acute ICH.

Tumorigenesis and instability both are the severe side-effects of iPSC transplantation. In this study, we have not observed any teratoma formation in all iPSCs-treated rats throughout the period of experiment. In addition, no other side-effect such as seizure or hyperpyrexia was observed during experimental period. However, these safety concerns related with iPSC transplantation should be carefully evaluated in future investigations with extended test periods [24]. Moreover, more studies are needed for further understanding of the properties and effects of engrafted iPSCs in animal models of human ICH before being used in clinical work [29, 33].

## Conclusions

These data support the hypothesis that the underlying mechanisms of the therapeutic effects of iPSC transplantation on experimental ICH may involve modulating inflammatory responses and exerting neuroprotective effects, which collaboratively contribute to the improvement in neurological and survival outcomes. Deciphering the properties and effects of iPSC transplantation on experimental ICH provides fundamental insights into the therapeutic mechanisms, which in turn reveal reliable strategies to make the iPSC treatment increasingly efficient and safe. Thus, these favorable insights into the immunomodulatory and therapeutic properties of iPSCs may further facilitate their autologous transplantation becoming a valuable strategy for treating hemorrhagic stroke.
